# Verifying origin claims on dairy products using stable isotope ratio analysis and random forest classification

**DOI:** 10.1016/j.fochx.2023.100858

**Published:** 2023-09-03

**Authors:** Roisin O' Sullivan, Raquel Cama-Moncunill, Michael Salter-Townshend, Olaf Schmidt, Frank J. Monahan

**Affiliations:** a UCD School of Agriculture and Food Science, University College Dublin, Belfield, Dublin D04 V1W8, Ireland; b UCD School of Mathematics and Statistics, University College Dublin, Belfield, Dublin D04 V1W8, Ireland

**Keywords:** Authenticity, IRMS, Geographic, Milk, Butter, Cheese

## Abstract

Scientifically underpinning geographic origin claims will improve consumer trust in food labels. Stable isotope ratio analysis (SIRA) is an analytical technique that supports origin verification of food products based on naturally occurring differences in isotopic compositions. SIRA of five relevant elements (C, H, N, O, S) was conducted on casein isolated from butter (n = 60), cheese (n = 96), and whole milk powder (WMP) (n = 41). Samples were divided into four geographic regions based on their commercial origin: Ireland (n = 79), Europe (n = 67), Australasia (n = 29) and USA (n = 22). A random forest machine learning model built using δ^13^C, δ^2^H, δ^15^N, δ^18^O and δ^34^S values of all products (n = 197) accurately (88% model accuracy rate) predicted the region of origin with class accuracy of 95% for Irish, 84% for European, 71% for Australasia, and 94% for US products.

## Highlights

• Isotope ratio analysis detects differences in geographic origin of dairy products.
• C, N and S isotope ratios of casein were the most important regional discriminators.
• Random forest model classified products by region with 88% accuracy.

## Introduction

Interest in label claims relating to the origin of foods has risen as more consumers aim to purchase with environmental and sustainability values in mind (Petrescu et al., 2020). The origin of a food product is often considered an indicator of food quality (Fraser & Balcombe, 2018) and consumers who are more attentive and willing to pay a premium for higher quality foods are interested in the origin of such foods (Merlino et al., 2022). While supply chain issues arise from climate change and unpredictable global health and political crises, there is, at the same time, a lack of consistent traceability practices associated with the import and export of dairy products. Therefore, science-based origin-confirming solutions must be developed to ensure a holistic view of the dairy supply chain.

Consumers are now knowledgeable about the differences between locally supplied and imported dairy products, especially in relation to quality, sustainability, animal welfare and market availability (Merlino et al., 2022), and therefore have particular interest in the origin of the products they purchase. Regarding premium products such as specialty cheeses, consumers are willing to pay more for high-quality products where geographic indications are more evident (Slade et al., 2019). EU regulations have been developed to meet consumer demand, reinforcing the importance of the ability to differentiate between the production origin of a food product and the primary origin of food ingredients. For example, Regulation (EU) 2018/775 states that “the country of origin or the place of provenance of a primary ingredient which is not the same as the given country of origin or the given place of provenance of the food shall be given”.

Various traceability methods have been used to support the verification of authenticity of dairy products (Ye et al., 2023), for example, paper-based traceability (Lo Bello et al., 2005), DNA and chromatography based authentication (Peres et al., 2007), and trace elemental analysis (Danezis et al., 2020). There continues to be a need to develop authentication tools for the dairy industry that are reliable, reproducible, and mechanistically understood.

Stable isotope ratio analysis (SIRA) is an analytical method that meets all of these criteria and has been used to support the geographic authentication of a variety of food products including wine (Christoph et al., 2015), meat (Bontempo et al., 2023, Monahan et al., 2018), milk and dairy products (Gregorcic et al., 2020, O'Sullivan et al., 2020). SIRA has become an effective origin-confirming tool for the wine industry where isotopic results must be compared to representative samples from official wine databanks such as that set out by the European Commission in Regulation (EC) No 479/2008. There is potential for a similar databank to be developed to underpin geographic origin claims made on dairy product labels.

In several studies, SIRA has been conducted on individual dairy products to authenticate their origin (Bontempo et al., 2011, Camin et al., 2012, Camin et al., 2004, Crittenden et al., 2007, Pianezze et al., 2020, Potocnik et al., 2020, Rogers et al., 2023). However, no study has examined the potential to predict the origin of different dairy products from a large, international sample set. The current study builds on previous work by using casein as a common component isolated from three different dairy products and assembling a large sample set which is representative of international commercial dairy products. This could provide evidence of a stable isotope signature associated with all dairy products produced within an individual country or a geographically defined region. In this study we take Ireland as an example and seek to answer the question: can Irish dairy products be identified from dairy products of a different origin using SIRA?

The objectives of this study were to (i) conduct SIRA on casein isolated from different dairy products, (ii) develop a general classification model based on casein SIRA values for predicting geographical origin with high accuracy that could be applied to all dairy products and (iii) evaluate which SIRA values (δ^13^C, δ^2^H, δ^15^N, δ^18^O or δ^34^S) are the most useful for the prediction of geographical origin.

## Materials and methods

### Sampling

Commercial samples (n = 197) of butter (n = 60), hard cheeses (n = 96), and whole milk powder (WMP) (n = 41), from different countries, were sourced from dairy industry sources and through retailers. These countries were grouped into four regions as follows: Ireland (Irish samples, n = 79); Europe (excluding Irish) (Austrian (n = 1), Danish (n = 13), Dutch (n = 15), French (n = 18), Italian (n = 5), Polish (n = 1), Spanish (n = 9), Swiss (n = 4), and UK (n = 1) samples, n = 67); Australasia (Australian (n = 3), Chinese (n = 3), and New Zealand (n = 23) samples, n = 29); the USA (US samples, n = 22). For this paper, we use the geographical term Australasia loosely by including 3 Chinese samples.

### Sample preparation

#### Reagents

Diethyl ether and isopropanol were purchased from VWR Chemicals (Ballycoolin, Dublin, Ireland). Petroleum spirit was purchased from Sigma-Aldrich (Wicklow, Ireland). Heptane was purchased from Lennox Laboratory Supplies (Dublin, Ireland). Hydrochloric acid (HCl; Laboratory grade) was purchased from Fisher Scientific (Loughborough, United Kingdom). Deionized water was obtained from a Millipore Elix 15 water purification system (Merck Millipore, Darmstadt, Germany).

#### Casein isolation

Casein was isolated from butter, cheese and WMP according to the methods described recently in O'Sullivan et al. (2023). In brief, butter samples were melted, cheese samples were grated and freeze-dried (Buchi Lyovapor L-200) and WMP samples were reconstituted in water, prior to lipid removal by solvent (petroleum spirit: diethyl ether (2:1, v/v)) extraction and centrifugation (Beckman Coulter Avanti J-E Centrifuge). Casein was precipitated from the defatted samples by acidification to pH 4.6, washed, separated by centrifugation, and then freeze-dried. For butter and WMP samples, an additional solvent lipid extraction step using heptane: isopropanol (3:2, v/v) was necessary to isolate a high purity casein sample. The casein fraction was freeze-dried and stored in a desiccator prior to SIRA.

### Stable isotope ratio analysis

Stable Isotope Ratio Analysis (SIRA) of C, H, N, O and S was conducted by Elemental Analysis–Isotope Ratio Mass Spectrometry (EA-IRMS) using a Europa Scientific elemental analyser and 20–20 IRMS according to the methods and reference materials described in O'Sullivan et al. (2023). All samples were analysed at IsoAnalytical Ltd. (Crewe, UK). Briefly, carbon dioxide and nitrogen gas were measured simultaneously for δ^13^C and δ^15^N values, while sulphur dioxide gas were measured separately for δ^34^S. For δ^18^O, samples along with calibration standards were comparatively equilibrated for 7 days prior to analysis and carbon monoxide and nitrogen gases were separated on a GC column packed with a molecular sieve at 45 °C. The analysis involved a batch process by which a reference was analysed followed by several samples and then another reference. The main standard reference materials used were soy protein (δ^13^C_V-PDB_ = −25.22‰, δ^15^N_AIR_ = 0.99‰), barium sulphate (δ^34^S_V-CDT_ = +20.33‰), and cane sugar (δ^18^O_V-SMOW_ = 35.23‰) for δ^13^C, δ^15^N, δ^34^S, and δ^18^O, respectively. These standards are calibrated and traceable to IAEA inter-laboratory standards. In addition, check samples were run during batch analysis for quality control purposes. Based on these, the analytical precision (SD) in the present study was 0.04‰ for δ^13^C (n = 18 replicates, soy protein), 0.02‰ for δ^15^N (n = 18 replicates, soy protein), 0.08‰ for δ^34^S (n = 24 replicates, barium sulphate), 0.12‰ for δ^18^O (n = 20 replicates, cane sugar) and 0.45‰ for δ^2^H (n = 28 replicates, mineral oil). For δ^2^H, samples and references were weighed (1.0 ± 0.1 mg) into silver capsules and comparatively equilibrated with moisture in the laboratory air for 14 days prior to analysis. Five non-exchangeable hydrogen standards were comparatively equilibrated and analysed alongside samples as controls. Of these, three standards used for calibration were human hair (non-exchangeable δ^2^H_V-SMOW_ = −44.4‰), caribou hoof (non-exchangeable δ^2^H_V-SMOW_ = −157.0‰) and kudu horn (non-exchangeable δ^2^H_V-SMOW_ = −35.3‰). The further two standards, measured as quality control check samples, were human hair (non-exchangeable δ^2^H_V-SMOW_ = −72.9‰) and casein (non-exchangeable δ^2^H_V-SMOW_ = −113.4‰). Analytical precision was 0.4‰ for δ^2^H (mineral oil).

### Data analysis

The ability to classify dairy products according to their geographical origin based on their stable isotope profiles was evaluated by a random forest approach. Random forest is a supervised machine learning algorithm that builds several decision trees based on the dataset to create a highly accurate prediction of classification. A random forest model was built using the commercial samples (n = 198) detailed in Section 2.1.

Data was split into training and test datasets using a 0.75 split ratio (75% of data was used to train the model and 25% was used to test the model). The training dataset was used to develop a random forest model with all five stable isotope ratio values (δ^13^C, δ^15^N, δ^18^O, δ^2^H and δ^34^S) and the most important values determined by the Gini Index (δ^13^C, δ^15^N and δ^34^S). All random forest models were developed using 500 trees with two variables tried at each split. The performance of the models was first evaluated with the training data set using the out-of-bag (OOB) observations resulting from the bootstrapping (i.e., observations not selected for building the model) and secondly, with the test set. For both validations, the following performance indicators were computed and compared: number of samples correctly classified, class accuracy and overall accuracy. This procedure was repeated three times, where different random training/tests splits were applied to evaluate the performance of the model. Results are expressed as the average class predictions obtained from each of these three data split combinations. This ensured that any differences found were not simply due to random variation. The contribution/importance of each variable was assessed by the mean decrease in Gini coefficient (Gini index) which measures the contribution of each variable to each split in the random forest model. Data analysis was conducted in R studio (Rstudio team, 2022).

## Results & discussion

### SIRA

Descriptive statistics for each individual country and for each geographical region of interest are shown in Table 1. δ^13^C values varied widely between countries with most negative values obtained for the Irish (−26.36‰), New Zealand (−26.3‰), Australian (−26.2‰) and UK (−26.1‰) samples (Table 1(a)). Least negative δ^13^C values were obtained for the US (−18.4‰), Chinese (−19.6‰) and Austrian (−19.8‰) samples. δ^13^C values vary between plants with differing photosynthetic pathways (Marshall et al., 2007). C3 plants such as barley, sugar beet and perennial ryegrass (using the Calvin cycle photosynthetic pathway) discriminate more against ^13^CO_2_ in favour of ^12^CO_2_ than do C4 plants such as maize, tropical grasses and sugarcane (using the Hatch-Slack cycle photosynthetic pathway); therefore, the ratio of ^13^C/^12^C in the resultant sugar molecules is lower in C3 plants (δ^13^C values between −35 and −21‰) compared to C4 plants (δ^13^C values between −14 and −10‰) (Boutton, 1991, Ehleringer, 1991, Smith and Epstein, 1971). This is particularly apparent when maize is included in the diet, with Camin et al. (2008) identifying a shift of 0.7‰ to 1‰ in the δ^13^C values of milk casein with each 10% increase in maize content in the diet of cows.

**Table 1:** Mean ± standard deviation δ^13^C, δ^15^N, δ^18^O, δ^2^H and δ^34^S values of all products (a) by country and (b) by region.

**Country**	**n =**	**δ^15^N**	**δ^13^C**	**δ^34^S**	**δ^2^H**	**δ^18^O**
(a) **By Country**						
Australia	3	6.15 ± 0.44	−26.23 ± 0.69	8.74 ± 1.40	−91.45 ± 3.15	16.36 ± 1.36
Austria	1	5.44	−19.82	2.69	−97.62	14.64
China	3	4.35 ± 1.02	−19.62 ± 3.77	5.69 ± 2.58	−100.71 ± 9.05	11.04 ± 2.22
Denmark	13	5.22 ± 0.38	−23.54 ± 1.74	5.05 ± 0.42	−103.43 ± 4.30	12.38 ± 0.76
France	18	5.16 ± 0.70	−22.89 ± 2.23	4.99 ± 1.55	−98.67 ± 8.73	12.86 ± 2.17
Ireland	79	6.66 ± 0.52	−26.36 ± 1.21	6.68 ± 1.24	−99.27 ± 7.74	13.89 ± 1.80
Italy	5	5.85 ± 0.85	−24.15 ± 1.03	4.57 ± 3.56	−97.07 ± 7.59	13.73 ± 3.50
Netherlands	15	6.32 ± 0.70	−23.41 ± 1.26	3.57 ± 0.77	−98.85 ± 8.74	12.74 ± 1.84
New Zealand	23	5.61 ± 0.55	−26.26 ± 1.96	8.17 ± 1.84	−97.92 ± 9.97	14.17 ± 1.34
Poland	1	5.84	−22.49	1.51	−104.94	10.25
Spain	9	5.6 ± 0.50	−21.38 ± 1.25	5.16 ± 1.83	−98.69 ± 4.78	12.52 ± 0.89
Switzerland	4	5.54 ± 0.72	−25.49 ± 0.77	3.99 ± 1.89	−115.13 ± 3.17	9.89 ± 1.37
UK	1	7.21	−26.06	4.48	−104.49	12.10
USA	22	5.57 ± 0.28	−18.40 ± 1.36	0.34 ± 1.97	−107.94 ± 10.32	10.22 ± 1.88

**Region**	**n =**	**δ^15^N**	**δ^13^C**	**δ^34^S**	**δ^2^H**	**δ^18^O**

(b) **By Region**						
Ireland	79	6.66 ± 0.52	−26.36 ± 1.21	6.68 ± 1.24	−99.27 ± 7.74	13.89 ± 1.80
Europe (excluding Ireland)	67	5.61 ± 0.79	−23.18 ± 1.94	4.52 ± 1.73	−100.67 ± 8.19	12.56 ± 2.00
Australasia	29	5.53 ± 0.75	−25.57 ± 2.94	7.97 ± 2.05	−97.52 ± 9.65	14.08 ± 1.90
USA	22	5.57 ± 0.28	−18.40 ± 1.36	0.34 ± 1.97	−107.94 ± 10.32	10.22 ± 1.88

δ^13^C values differ between dairy products manufactured from milk-producing animals fed different diets (Camin et al., 2008). The diet of a milk-producing animal often depends on where an animal was raised and what type of feedstuff is most economically suitable in that region. For pasture-based dairy production systems, climatic conditions influence the types of plants grown in a region, with C4 plants more commonly grown in tropical and subtropical climates with low precipitation levels and C3 plants more commonly grown in more temperate climates with higher precipitation levels (Still et al., 2003). Reflecting this general observation, in this study, δ^13^C values were more negative in Irish, New Zealand and Australian dairy products (Table 1(a)) indicating a more C3 plant-based diet for milk producing animals from these regions. This is consistent with the pasture-based dairy production systems associated with these countries based on their climatic conditions (Hurtado-Uria et al., 2013, Wales and Kolver, 2017). In contrast, δ^13^C values were least negative in Chinese and US dairy products (Table 1(a)) suggesting a more C4 plant-based diet for milk-producing animals, consistent with the concentrate-based (maize grain) dairy production systems associated with these regions (von Keyserlingk et al., 2013, Wang et al., 2022).

Highest δ^15^N values were obtained for the UK (7.2‰), Irish (6.7‰), Dutch (6.3‰) and Australian (6.2‰) samples. Lowest δ^15^N values were obtained for the Chinese (4.4‰) and French (5.2‰) samples. δ^15^N values are also associated with the diet of a milk-producing animal and reflect the N source of the plant making up the diet. Plant N sources are either atmospheric N for legumes (Gentili & Huss-Danell, 2019) or soil nitrogen for all other plants. The soil N composition in turn is influenced by agricultural practices such as fertiliser application (Gurmesa et al., 2017). For temperate pasture-based dairy production systems, there is a large reliance on inorganic fertilizers to increase the yield and quality of forage crops (Delaby et al., 2020). The high use of mineral N fertilizers in intensive pasturing systems results in open N cycles, which are usually associated with large N losses (through ammonia volatilisation and NO_x_ gas production) to the wider environment and a gradual ^15^N enrichment of the soil N pool (Harris et al., 2022, Mudge et al., 2013). Consequently, intensive pasture-based dairy production systems generally are associated with milk having more positive δ^15^N values (Dong et al., 2017, O'Sullivan et al., 2020). In the current study, δ^15^N values were high in Irish and Australian samples (Table 1(a)), indicating a more highly pasture-based dairy production system in these countries, supporting the δ^13^C results, which indicated the same. Lower δ^15^N values were detected in Chinese and some European samples (Table 1(a)) suggesting a higher level of cereal feedstuffs in their dairy production systems.

For δ^34^S, values were highest for Australian (8.7‰), New Zealand (8.2‰), and Irish (6.7‰) samples. δ^34^S values were lowest in US samples (0.3‰), followed by the Polish (1.5‰), Austrian (2.7‰) and Dutch samples (3.6‰). δ^34^S values reflect the geology of a region (Chivas et al., 1991) and are also influenced by the “sea-spray” effect leading to enriched δ^34^S values of soil close to coastlines. The existence and substantial extent of this sea-spray effect was documented by Zazzo et al. (2011) who showed that coastal δ^34^S values of sheep wool in Ireland were much higher closer to the coast and gradually declined moving further inland (>100 km). In the present study, the exact location of where the milk-producing animals were raised and, thus, the milk used to produce the products, is unknown; therefore, we cannot assume that there was a sea-spray effect. However, inland Europe and US samples had lower δ^34^S values compared to island countries and those with large coastlines such as Ireland and New Zealand (Table 1(a)), probably indicating a continental versus maritime effect consistent with previous studies on δ^34^S values of milk samples (Crittenden et al., 2007, O'Sullivan et al., 2020).

**Fig. 1:**
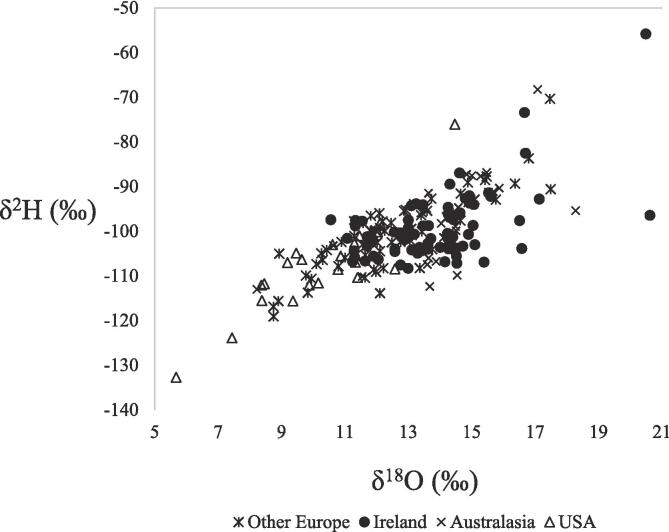
Plot of δ^2^H and δ^18^O values for all countries and products (n = 197) from Ireland, Australasia, Europe (excluding Irish) and USA region groups (r^2^ = 0.54).

Generally, when δ^18^O values were higher, δ^2^H values were also less negative (r^2^ = 0.54) (Fig. 1), with Australian δ^18^O and δ^2^H values of 16.4‰ and −91.5‰, respectively, and δ^18^O and δ^2^H values of 14.6‰ and −97.6‰, respectively, for the Austrian sample. Conversely, when δ^18^O values were lower, δ^2^H values were more negative with δ^18^O and δ^2^H values of 9.9‰ and −115.1‰ for Swiss, and 10.2‰ and −107.9‰ for US samples, respectively. δ^18^O and δ^2^H values of milk vary depending on an animal’s water source, which in turn reflects the precipitation δ^18^O and δ^2^H values of the region if the source is rain-fed (Abeni et al., 2015, Marshall et al., 2007). Precipitation δ^18^O and δ^2^H values are influenced by seasonality (Abeni et al., 2015, Marshall et al., 2007), latitude (Bowen et al., 2005), altitude (Gonfiantini et al., 2001) and continental effects (Bowen, 2010). Precipitation δ^18^O and δ^2^H values are closely related on the global meteoric water line (GMWL) associating less negative δ^2^H values with more positive δ^18^O values (Marshall et al., 2007). This pattern was established in Austrian, Australian, Swiss and US dairy products (Table 1(a), Fig. 1) in this study. Spatial mapping of precipitation isotope values (isoscapes) have been developed and are regularly updated recording δ^18^O and δ^2^H values at a global scale (Bowen, 2010). Based on this data, the effect of latitude has been reported with precipitation δ^18^O and δ^2^H values becoming more negative with increasing latitude (i.e. moving further from the equator) (Bowen et al., 2005). In the current study, δ^18^O and δ^2^H values were less negative for low latitude countries such as Spain and Italy when compared to countries further from the equator such as Denmark and Switzerland (Table 1(a)). However, for Australia and New Zealand, which would be considered high latitude countries, δ^18^O and δ^2^H values reflected those of lower latitude countries (Table 1(a)). Given that the samples used in this study were sourced commercially, the time of year when the samples were produced is unknown; however previous studies have reported a seasonal influence on δ^18^O and δ^2^H values with more negative values in winter and less negative in summer (Abeni et al., 2015, Kornexl et al., 1997) which may have masked the latitude effect.

Differences between milk and precipitation δ^18^O and δ^2^H values due to fractionation and enrichment that occur within the milk producing animal have also been reported (Boito et al., 2021). For example, Gregorcic et al. (2020) noted that milk water was enriched in ^18^O isotopes compared to animal drinking water. Seasonal and geographic changes in δ^18^O values of forage plants and thus, an animals’ body tissues, have been linked to evapotranspiration (Barbour, 2007) with higher evapotranspiration rates in dry zones leading to enriched δ^18^O values in plants and milk derived from milk-producing animals consuming those plants (Kalpage et al., 2022, Kornexl et al., 1997). Factors such as breed Bryant & Froelich (1995) and reproductive stage can influence δ^18^O and δ^2^H values and result in differentiation between milk and precipitation δ^18^O and δ^2^H values. Another, complicating factor is the consumption of water from ground water sources as opposed to precipitation. In a given location, there can be isotopic differences between surface and ground waters, caused by time lags between precipitation events and groundwater recharge, or by the existence of very old groundwaters such as Pleistocene waters in Britain (e.g. Darling et al. (2003)). Therefore, there is a need to develop isoscapes and databanks for stable isotope ratio values of dairy products specifically since precipitation isoscapes cannot be relied upon to support the verification of origin label claims on dairy products.

In previous papers, focus has often been placed on the power of an individual stable isotope ratio to identify differences between samples. For example, Pianezze et al. (2020) discriminated between Italian cheeses under the Protected Designation of Origin (PDO) label from different regions using δ^34^S alone, Gregorcic et al. (2020) identified differences in δ^18^O and δ^2^H values of Slovenian milk from different regions, and Rogers et al. (2023) used all five stable isotope ratio values to reliably discriminate New Zealand butter from Chinese, USA and European labelled products. In the present study, casein was isolated from butter, cheese and WMP products to enable meaningful comparison of different dairy products based on origin alone. The factors affecting stable isotope ratios were reflected in the influence of the dairy production system of a region on the δ^13^C and δ^15^N values of dairy products, with more negative δ^13^C and more positive δ^15^N values indicating a pasture-based production system. However, it should be noted that if a pasture has a high legume content the δ^15^N value may be less positive (Bai et al., 2009). Additionally, the potential influence of land mass and geographic location on δ^34^S, δ^18^O and δ^2^H values was noted. However, the power of this study lies in the combination all five stable isotope ratio values (δ^13^C, δ^15^N, δ^18^O, δ^2^H and δ^34^S) allowing several influencing factors to be considered and a more complete isotopic profile of the casein isolated from the dairy products to be developed.

### Random forest and Gini index

The random forest model described in Section 2.4 using all five stable isotope ratio values (δ^13^C, δ^15^N, δ^18^O, δ^2^H and δ^34^S) had an overall accuracy of 87.8% (Table 2). Based on the test data, Irish dairy products had the greatest class accuracy (94.7%), followed by US (94.4%), European (excluding Irish) (84.3%) and Australasia dairy products which had the lowest class accuracy (71.4%) (Table 2). Although the samples included in the current study did not cover Eastern and Central Europe sufficiently well (one sample from Poland and none from Germany), and we obtained only one sample from the UK, future studies should add to this database, which may improve the validity of the model.

**Table 2:** Random Forest classification results obtained from out-of-bag (OOB) cross validation and independent validation for four groups consisting of samples from Ireland, Europe (excluding Irish), Australasia, and USA region groups using δ^13^C, δ^2^H, δ^15^N, δ^18^O and δ^34^S values.

		Predictions					
		n =	Ireland	Europe (excluding Irish)	Australasia	USA	Class Accuracy (%)
**OOB**	Ireland	58	52.3	3.7	2.0	0.0	90.2
	Europe (excluding Irish)	50	4.0	44.3	0.67	1.0	88.7
	Australasia	21	4.0	2.67	12.3	2.0	58.7
	USA	16	0.0	1.7	0.0	14.3	89.6
**Test**		n =	Ireland	Europe (excluding Irish)	Australasia	USA	Class Accuracy (%)
	Ireland	19	18.0	1.0	0.0	0.0	94.7
	Europe (excluding Irish)	17	2.0	14.3	0.0	0.7	84.3
	Australasia	7	0.3	1.7	5.0	0.0	71.4
	USA	6	0.0	0.3	0.0	5.7	94.4
**Model accuracy (%)**	**87.8**						

* built using three test data sets resulting from randomly splitting the data into training and tests sets three times.

**Fig. 2:**
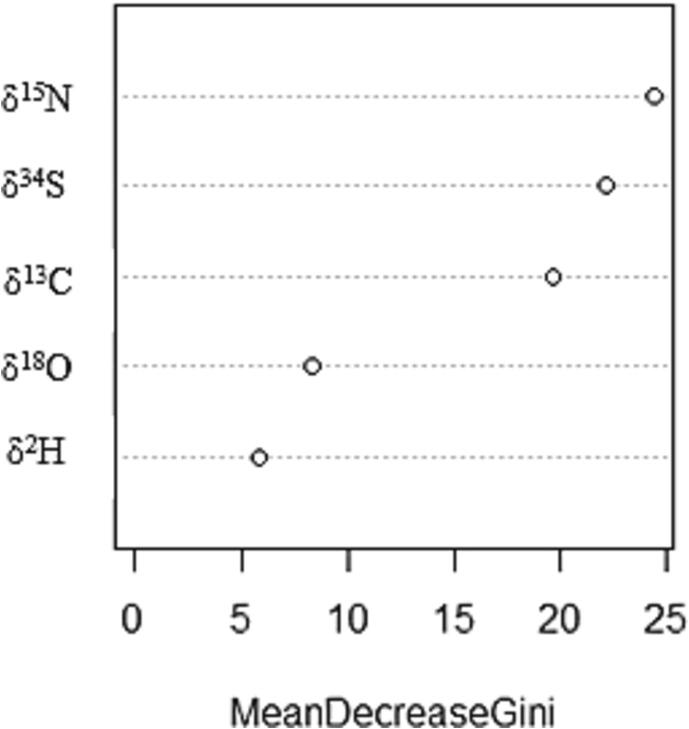
Variable importance plot as measured by Gini index using Random Forest machine learning model.

The Gini index is a measure of variance which describes the quality of the ability of a variable to classify a sample set within the random forest model (Han et al., 2016). The importance of each element’s stable isotope ratio in distinguishing between samples of different geographic regions was ranked using the mean decrease in Gini coefficient; the higher the number the more important that variable was in classifying the dataset based on region of origin (Fig. 2). Each element’s stable isotope ratio value (δ^13^C, δ^15^N, δ^18^O, δ^2^H and δ^34^S) provides specific background information about the geographic origin and production background of the milk-producing animal (Section 3.1). In the present dataset, δ^13^C, δ^34^S, and δ^15^N values were highly important when classifying samples while, δ^18^O and δ^2^H values were less useful (Fig. 2). Therefore, a random forest model was built using the three most important values (δ^13^C, δ^15^N and δ^34^S) which had an overall accuracy of 84.4% (Table 3). Based on the test data, Irish dairy products had the greatest class accuracy (94.7%), followed by US (94.4%), European (excluding Irish) (76.5%) and Australasia dairy products which had the lowest class accuracy (66.7%) (Table 3). Since δ^13^C and δ^15^N values are known to relate to a dairy animal’s feeding regime (Section 3.1), this indicates that the feedstuffs consumed in a particular geographic region strongly influence the ability of the model to predict region of origin of a dairy product. However, when δ^18^O and δ^2^H values were included in the random forest prediction model, the overall model accuracy was slightly better than when they were excluded (Table 2, Table 3). This demonstrates that using all five stable isotopes in combination allowed the development of a successful prediction model which agrees with previous studies whereby multi element analysis has been determined as most effective at identifying differences between food samples (Ng et al., 2021, Won et al., 2021).

**Table 3:** Random Forest classification results obtained from out-of-bag (OOB) cross validation and independent validation for four groups consisting of samples from Ireland, Europe (excluding Irish), Australasia, and USA region groups using δ^13^C, δ^15^N, and δ^34^S values.

		Predictions					
		n =	Ireland	Europe (excluding Irish)	Australasia	USA	Class Accuracy (%)
**OOB**	Ireland	58	50.3	5.3	2.3	0.0	86.8
	Europe (excluding Irish)	50	4.0	44.0	1.0	1.0	88.0
	Australasia	21	3.0	3.3	12.7	2.0	60.3
	USA	16	0.0	1.0	0.0	15.0	93.8
**Test**		n =	Ireland	Europe (excluding Irish)	Australasia	USA	Class Accuracy (%)
	Ireland	19	18.0	1.0	0.0	0.0	94.7
	Europe (excluding Irish)	17	2.7	13.0	0.7	0.7	76.5
	Australasia	7	0.0	2.3	4.7	0.0	66.7
	USA	6	0.0	0.3	0.0	5.7	94.4
**Model accuracy (%)**	**84.4**						

* built using three test data sets resulting from randomly splitting the data into training and tests sets three times.

SIRA is an analytical method that is reproducible with results which can be explained mechanistically, making it an advantageous method for assessing the authenticity of food products, particularly foods produced from animal sources (Camin et al., 2016). The main factors underlying present results have been discussed briefly in Section 3.1. In this study, the random forest prediction model detected the highest level of class accuracy for Irish dairy products (Table 2). Ireland is a unique dairy production location with consistent precipitation and moderate temperatures suitable for grass growth and, thus, dairy animals can graze outdoors for up to 300 days a year (O'Donovan et al., 2011). The lowest class accuracy was for the Australasia region (Table 2). Dairy production systems in New Zealand and Australia are generally considered to be pasture-based (Moscovici Joubran et al., 2021) whereas, dairy production systems in China are less well defined (Fuller et al., 2006). Therefore, the vast and loosely defined geographic scale within the region and varying dairy production and consequential feeding systems likely influenced the reduced ability to classify samples in the Australasia region.

Previous studies have demonstrated the ability for SIRA to be used as an origin confirming tool. For example, Luo et al. (2016) examined the use of δ^13^C, δ^15^N, δ^18^O and δ^2^H values in determining the region of origin of 30 milk samples from Australia and New Zealand (n = 9), Germany and France (n = 6), the US (n = 7) and China (n = 8) with significant differences (p < 0.01) between δ^13^C and δ^15^N values of milk protein and δ^18^O values of milk water when samples were split into four regions of origin. This study provided initial evidence of the potential for stable isotope ratio values to be used as an origin determination tool for milk. The study by Perini et al. (2022) investigated the use δ^2^H values of lactose and δ^18^O values of bulk milk as a geographic indicator of milk in Europe. This paper provided evidence of the need to isolate a particular component from products in order to compare different dairy products. Having multiple elements and multiple products, or product components, makes it possible to apply multivariate statistical approaches which has been shown to be useful in previous large scale stable isotope studies such as Bontempo et al. (2023). In Bontempo et al. (2023), 227 European beef samples from eight countries were divided into three groups (samples from inland areas sited at relatively low latitudes, where the animals received a mixed diet; samples from coastal areas sited at relatively low latitudes, where the animals received a C4-based diet; and samples from coastal areas sited at higher latitudes compared to the other groups, where the animals received a C3-based diet) and δ^13^C, δ^15^N, δ^2^H and δ^34^S values were used to identify differences between groups. This approach was advanced in the current study by using a random forest model.

## Conclusions

A multivariate random forest model including all five stable isotope ratio values (δ^13^C, δ^15^N, δ^18^O, δ^2^H and δ^34^S) was used successfully to discriminate between different regions of origin of dairy product (cheese, butter and WMP) samples with a high level of accuracy (88%). This study provides a unique and highly precise initial database and prediction model which has the potential to inform a future stable isotope databank for dairy products internationally that could be used as a reference when supporting the verification of origin claims on dairy products. This study also provides evidence of the potential to produce a “dairy stable isotope signature” for individual countries or regions which can be used to prevent fraudulent mislabeling of sample origins and enhance traceability systems within the dairy industry.

## CRediT authorship contribution statement

**Roisin O' Sullivan:** Formal analysis, Investigation, Data curation, Writing – original draft, Writing – review & editing, Visualization. **Raquel Cama-Moncunill:** Methodology, Software, Data curation, Writing – review & editing. **Michael Salter-Townshend:** Methodology, Writing – review & editing. **Olaf Schmidt:** Conceptualization, Methodology, Writing – review & editing, Supervision. **Frank J. Monahan:** Conceptualization, Methodology, Resources, Writing – review & editing, Supervision, Project administration, Funding acquisition.

## Declaration of Competing Interest

The authors declare that they have no known competing financial interests or personal relationships that could have appeared to influence the work reported in this paper.

## Data Availability

Data will be made available on request.
